# Ion Chromatographic Fingerprinting of STC-1 Cellular Response for Taste Sensing

**DOI:** 10.3390/s19051062

**Published:** 2019-03-02

**Authors:** Marcin Zabadaj, Aleksandra Szuplewska, Maria Balcerzak, Michał Chudy, Patrycja Ciosek-Skibińska

**Affiliations:** 1 The Chair of Medical Biotechnology, Faculty of Chemistry, Warsaw University of Technology, Noakowskiego 3, 00-664 Warsaw, Poland; mzabaj@ch.pw.edu.pl (M.Z.); aszuplewska@ch.pw.edu.pl (A.S.); chudziak@ch.pw.edu.pl (M.C.); 2 Faculty of Chemistry, Warsaw University of Technology, Noakowskiego 3, 00-664 Warsaw, Poland; mbal@ch.pw.edu.pl

**Keywords:** taste sensing, bioelectronic tongue, STC-1 cells, ion chromatography, chromatographic fingerprints, PLS-DA

## Abstract

Taste sensing is of great importance in both the pharmaceutical and foodstuff industries, and is currently mainly based on human sensory evaluation. Many approaches based on chemical sensors have been proposed, leading to the development of various electronic tongue systems. However, this approach is limited by the applied recognition methods, which do not consider natural receptors. Biorecognition elements such as taste receptor proteins or whole cells can be involved in the development of taste sensing biosensors usually equipped with various electrochemical transducers. Here, we propose a new approach: intestinal secretin tumor cell line (STC-1) chemosensory cells were applied for taste recognition, and their taste-specific cellular response was decoded from ion chromatographic fingerprints with the use of multivariate data processing by partial least squares discriminant analysis (PLS-DA). This approach could be useful for the development of various non-invasive taste sensing assays, as well as for studying taste transduction mechanisms in vitro.

## 1. Introduction

Gustation is one of the most meaningful physiological functions of mammalian organisms. This function greatly assists the choices of animals in terms of food nutrition, e.g., sweet represents precious energy sources and bitter indicates a wide range of toxic substances. The taste receptors expressed in animals’ tongue bud cells determine the animals’ taste perception abilities [1,2,3]. Until now, it has been challenging to mimic mammalian taste sensing, while it could be very useful for taste evaluation in both the pharmaceutical and foodstuff industries, where human panels could be replaced by so-called “electronic tongues” or “taste sensing systems”. Even though many efforts to elaborate such devices have been presented so far, and commercialized versions are available, it is still believed that such fingerprinting based on chemical sensor array responses is a quite distant analogy of mammalian gustation due to limited sensitivity and selectivity linked to insufficient mimicking of the complex and interconnected downstream signaling cascades involved in taste sensing [4,5,6,7,8].

Therefore, an alternative approach was proposed, based not on chemical sensors, but biological sensing elements, namely taste receptor cells (TRCs) [3,5,9,10,11]. TRCs are widely recognized as biological functional elements that may be applied for the evaluation of responses to basic taste stimuli. To date, there are two classes of cells available for the fabrication of bioanalytical tools, namely natural taste receptor cells with native taste receptors [12] and bioengineered taste receptor cells [13]. Bioengineered cells are a category of functional cells treated specially to be used as effective and sensitive elements for bioanalytical procedures. They can respond to the tastants or the specific compounds [14,15,16,17]. On the other hand, natural cells are derived from biological taste systems, which maintain their natural morphologies and properties. An example of the cells used as a tasting system model are chemosensory cells, isolated from, e.g., murine intestine [5,13,18,19]. Under physiological conditions, chemosensing is involved in the digestion and absorption of nutrients through neurohormonal control. Gut hormones mediate nutrient signals through the vagus nerve from the gut directly to the brain. The secretion of gut hormones is stimulated by dietary components through the activation of nutrient sensors.

Various cell-based systems or human taste receptors have been involved to datein the development of sensors or assays capable of detecting and discriminating tastes with human-like performance. Such bioelectronic tongues are comprised of various recognition elements: taste receptors or their ligand binding domains [20,21,22], whole cells [6,7,18], or tissues [10]. They were immobilized mainly at various electrochemical transducers, i.e., electrodes, field effect transistors (FETs) [7,21,22] or light-addressable potentiometric sensors (LAPS) [6,12]. An alternative was proposed in [23], where a quartz crystal microbalance (QCM) sensor was applied as a mass-sensing transducer for bitterness sensing.

In this study, we proposed a novel strategy of taste sensing. We monitored the responses of intestinal secretin tumor cell line (STC-1) cells, which are derived from the musculus gut and are sensitive to bitter, sweet, and umami taste [5,24], to five basic tastants via ion chromatographic fingerprinting (experimental workflow presented in the Supplementary Materials, Figure S1).

Ion chromatography (IC) presents a sequential multielemental determination capability, satisfying selectivity and reproducibility, a stable separation system, and speed of analysis, allowing the determination of biologically-important elements, e.g., calcium in the form of Ca^2+^ [19]. IC combined with conductometric detection, a well-established separation technique for the determination of a wide range of inorganic as well as organic ions, were applied to the cells to obtain ion chromatographic fingerprints of the cell culture media after treatment with five basic tastants: sodium chloride (salty taste), citric acid (sour taste), caffeine citrate (bitter taste), aspartame (sweet taste), and sodium glutamate (umami taste). Due to the secretion and fluxes of various ions [6,7,25], after the exposure of the cells to the tastants, we expected changes in the chromatographic profiles of the cell culture media. Ion chromatographic fingerprints, which are characteristic cellular responses to different taste stimuli, were the subject of classification used to reveal the possibility of taste identification. Such transduction of taste information, based on our best knowledge, is presented here for the first time, allowing for the possibility of non-invasive taste research and the study of taste mechanisms in vitro.

## 2. Materials and Methods

### 2.1. Samples Preparation

The STC-1 cell line (Mus musculus intestinal neuroendocrine tumor cells; American Type Culture Collection (ATCC)) was chosen as the research model. The cells were cultured in complete DMEM medium (Dulbecco’s Modified Eagle Medium, Sigma-Aldrich, Saint Louis, USA) supplemented with 10% (*v*/*v*) fetal bovine serum (FBS, Sigma-Aldrich), 1% of penicillin and streptomycin (Sigma-Aldrich) (*v*/*v*), and 1% of L-glutamine (Sigma-Aldrich) (*v*/*v*). Cells were maintained under 5% CO_2_ at 37 °C and 95% humidity and passaged once a week.

In order to prepare the samples for ion chromatographic analysis, the cells were seeded in the standard Petri dishes (60 mm of diameter, Nest) in a density of 2 × 10^6^ cells per dish. When the cells’ adhesion to the culturing surface was observed, the medium was removed and replaced with Dulbecco-modified phosphate buffered saline (DPBS: 200 mg/L KCl, 200 mg/L KH_2_PO_4_, 8 g/L NaCl, 2.16 g/L Na_2_HPO_4_·7H_2_O; Sigma-Aldrich) containing 1 mM of various tastants (sodium L-glutamate, caffeine citrate, aspartame, citric acid, sodium chloride; 10 mL per dish). Controls were carried out in the absence of the tested flavor substances (cell culture was incubated with the proper fresh buffer only). After 1 min of incubation under 5 % CO_2_ at 37 °C, the taste substance solutions were replaced with fresh DPBS (10 mL per dish). The samples of fresh DPBS were subsequently collected and frozen for further analysis. Directly before the injection on the column, samples were filtered using 0.22 µm nylon syringe filters (Metrohm) and C-18 microcolumns (Metrohm). Each sample was prepared in at least three independent replications.

### 2.2. Ion Chromatographic (IC) Analysis

The 761 Compact IC System, Metrohm AG (Herisau, Switzerland) with a conductometric detector, and the IC Net 2.3 Metrodata (Metrohm) software for data acquisition and evaluation of chromatograms were used. It was equipped with a Metrosep C 4 (150/4.0 (6.1050.420), Metrohm) column. The mobile phase consisted of 1.7 mM HNO_3_ (Fluka) and 0.7 mM dipicolinic acid (DPA, Fluka). Elution was performed in the isocratic mode for a total of 22 min. The flow rate was 900 µL per min, and the injection volume was 200 µL. The temperature of the column was 25 °C.

The method was validated in terms of its sensitivity, accuracy, precision, specificity, and linearity. The limits of quantification (LOQ) and limits of detection (LOD), respectively, a measure of a method’s sensitivity, were established based on the lowest spiked concentration. The relative standard deviation (RSD) calculated for each population of the results was below 11% [26].

### 2.3. STC-1 Cells’ Viability Evaluation

The biological activity of the tested tastants against the STC-1 cell line was evaluated by measuring the cells’ viability after their exposure to the studied substances in the concentration of 1 mM. Cell viability was evaluated using an MTT test—a colorimetric assay for assessing cell metabolic activity. NAD(P)H-dependent cellular oxidoreductase enzymes may, under defined conditions, reflect the percentage of cellular viability. These enzymes are capable of reducing the tetrazolium dye MTT 3-(4,5-dimethylthiazol-2-yl)-2,5-diphenyltetrazolium bromide to the insoluble in water media formazan, which is purple. Cells were seeded in the standard 96-well plates in a density of 1.5 × 104 cells per well. Subsequently, the cells were incubated to assure the adhesion to the culturing surface. Then, the medium was removed and replaced with proper fresh medium containing 1 mM of various tastants (100 μL per well). Controls were carried out in the absence of tested flavor substances (cell culture was incubated with the proper fresh medium only). The cell viability was measured after 24 h of incubation under 5% CO_2_ at 37 °C. The experiment was conducted at least twice independently. In order to verify the influence of the tested compounds on the cell culture, cells were treated with MTT (Sigma-Aldrich) solution (0.5 mg mL^-1^ in phosphate buffered saline (PBS) (Sigma; 100 µL per well). Cells were incubated with MTT solution for the next 4 h and protected from light. Then, the supernatant was carefully removed and the formed violet formazan crystals were dissolved in dimethyl sulfoxide (DMSO, Sigma-Aldrich; 100 µL per well). The absorbance of the formazan solutions was measured at 570 nm using a Multiwell Plate Reader (Biotek Cytation 3). The results were expressed as a percentage of viability in comparison to the control groups, according to the formula below.

Cells’ viability = *A*i/*A*c × 100%, (1)

where *A*i—average absorbance of tested group; *A*c—average absorbance of control group.

### 2.4. Data Analysis

The chemometric analysis was performed using the SOLO^®^ software (Eigenvector Research Inc., Manson, WA, USA) supplemented by in-house-written codes for Matlab (Mathworks Inc., Natick, MA, USA). Autoscalling and the SIMPLS regression algorithm were applied. Figures were generated using Origin ver. 9.0 (OriginLab Corporation, Northampton, MA, USA).

## 3. Results and Discussion

In this study, we monitored the responses of STC-1 cells (derived from musculus gut; sensitive to bitter, sweet and umami taste [5,24]) to five basic tastants by measuring the ionic cellular response in cell culture media, compared to controls. The STC-1 cells’ responses to tastant stimuli were determined using ion chromatography (IC) combined with conductometric detection, which is a well-established method in routine analysis for determination of a wide range of inorganic as well as organic ions.

### 3.1. Chromatograms of STC-1 Cellular Response Towards Taste Stimuli

**Figure 1:**
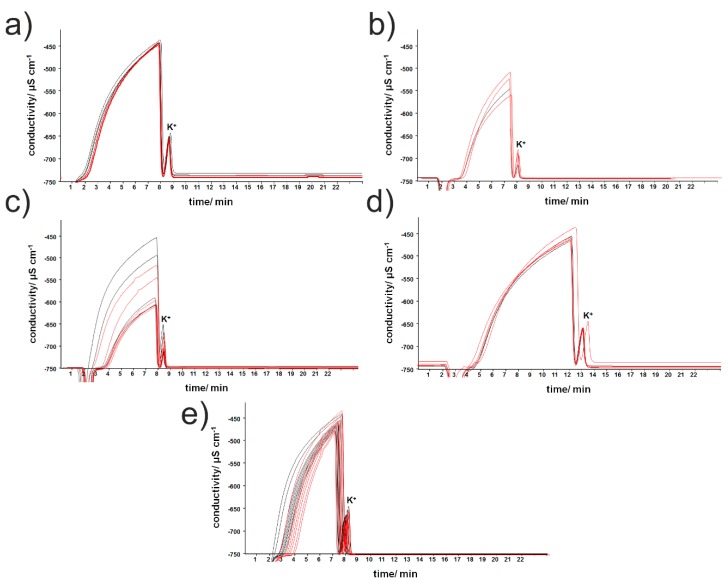
Examples of chromatograms (red) of Dulbecco-modified phosphate buffered saline (DPBS) added to intestinal secretin tumor cell line (STC-1) cells after the treatment with 1 mM solutions of: (**a**) aspartame, (**b**) citric acid, (**c**) caffeine citrate, (**d**) sodium L-glutamate, (**e**) sodium chloride. Black—control groups. Controls were carried out in the absence of taste substances.

The DPBS samples were collected after the cell cultures were exposed for 1 min to selected taste substances in the concentration of 1 mM. This concentration was chosen based on the fact that it corresponds to human recognition thresholds (from parts of millimols in the case of citric and acetic acid, through to a few millimols in the case of monosodium glutamate, to even 20–30 millimols in the case of sucrose or NaCl) [27]. The obtained chromatograms are shown in Figure 1. The wide, unseparated signal (between 2 and 8 min) as well as the potassium-cation peak (retention time circa 8 min) are visible on each chromatogram. The probable cause of the problems with the signal separation was the presence of the small organic molecules (e.g., amino acids, fatty acids) in sample matrix in too high concentrations, thereby obstructing their efficient removal using C-18 microcolumns and peak separation during further analysis [28]. Generally, a large dispersion of the results for the groups stimulated with tastants and controls was visible and smaller differences in the chromatogram shapes could be observed after the incubation of the cell cultures with aspartame only. In contrast, treating the cells with other taste substances caused changes in retention time and peak heights. Additionally, the dispersion of the results obtained for the samples after exposing the cell to sodium L-glutamate and sodium chloride precluded the easy contradistinction of those treated populations from the control groups (see Figure 3A). However, such preliminary observations must be treated carefully, and validation must be performed based on many replicates comparing with many controls to reveal possible statistically significant differences between the obtained chromatograms. The discrimination between the stimulated cells and controls is based on two effects simultaneously: the total variability of the signals, and variability within the groups. Therefore, our goal was to use chemometric calculations to recognize the potential alterations between the controls and stimulated groups on the basis of the whole shapes of the obtained chromatograms rather than based on individual, well separated peaks.

### 3.2. Viability Study

**Figure 2:**
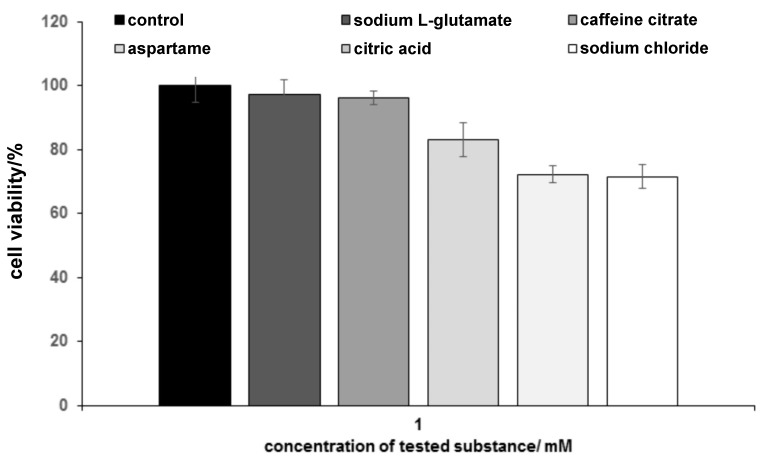
Cell viability after 24 h of exposure to 1 mM solutions of various taste substances (*n* = 3; *n*—number of independent experiments).

In order to ensure that the studied differences were not an artifact caused by cytotoxic effect in the studied cell cultures, the influence of tastants on the viability of chemosensory cells was also checked (see Figure 2). The cytotoxicity analysis was performed after 24 h of incubation of the cells with taste substances. The cell viability was determined using the standard MTT protocol. An MTT assay is based on tetrazolium salt thiazolyl blue reduction by the activity of the mitochondrial dehydrogenases present in living cells [29]. The purple product of the reaction is subsequently determined spectrophotometrically. As Figure 2 shows, the lack of obvious cytotoxic effects after 24 h of the exposure of chemosensory cells to the aspartame, sodium l-glutamate and citric acid could be noted. Statistically significant decrease after exposure to caffeine citrate and sodium chloride was observed. However, in these cases the cells maintained up to 70% viability after the 24 h incubation, which was sufficient to also regard these two tastants as non-toxic to the cells (the concentration of the tested compound can be described as non-toxic if the cell viability remains above 70% [30]). The obtained results suggest the higher sensitivity of STC-1 cells to caffeine citrate and sodium chloride as the probable cause of the observed effect. Nevertheless, the possible cytotoxic action of taste stimulants at millimolar concentrations and at the timespan of the taste experiment can be regarded as negligible.

### 3.3. Taste Recognition Using PLS-DA

**Figure 3:**
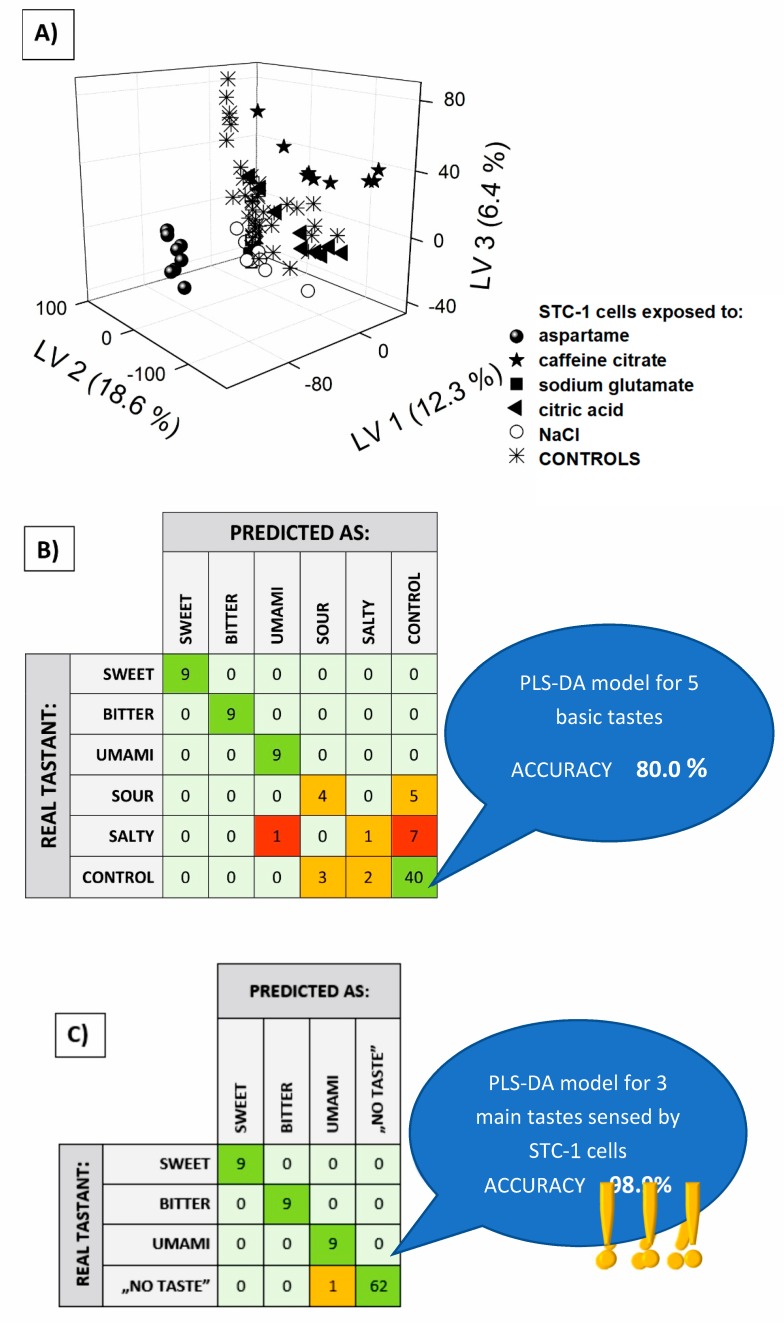
Partial least squares discriminant analysis (PLS-DA) classification of cellular responses towards five basic tastants: (**A**) 3D PLS-DA score plot of chromatographic fingerprints of the STC-1 cellular response; (**B**) and (**C**) confusion matrices for basic taste recognition.

Using the obtained chromatograms, quantitative data analysis was impossible, due to poor peak separation. Despite this, the obtained data can be useful, because full chromatograms are specific and unique fingerprints of the investigated samples, although they demand multivariate analysis for information decoding. We attempted ion chromatographic fingerprint classification by means of the partial least squares discriminant analysis (PLS-DA) method. The aim of the study was to discriminate media samples from the cell cultures exposed to the five basic tastants and from controls, therefore 0–1 coding was applied to assign the samples to six classes for the formation of a target matrix. The raw chromatographic curves, seen in Figure 1, were differentiated using the Savitzky–Golay algorithm, after careful optimization of the parameters (details in [31]) and the obtained vectors for each sample served as inputs for the PLS-DA (multivariate target matrix, PLS2 algorithm, number of LVs determined by minimization of the Root Mean Squared Error of Cross Validation RMSECV value, and cross-validation performed using venetian blinds). The result of the discrimination of the six classes was presented on a 3D-PLS score plot (Figure 3A). The best separation of clusters was visible for sweet and bitter taste (cells treated with aspartame and caffeine citrate, respectively), while the controls seemed to overlap for the other three tastes (cells exposed to NaCl, citric acid and sodium glutamate). A setailed investigation of this effect—discrimination between the controls and these three tastants, was made on the basis of one column classification for each taste. This revealed that the umami taste was easily discernable from the controls, while the resting ionic tastants (NaCl and citric acid) caused an insignificant change in the ion chromatographic fingerprints of cellular responses (Supplementary Materials, Figure S2). This finding is of great importance, since it correlates well with the natural taste sensing ability of STC-1 cells, which respond to sweet, bitter and umami stimuli [5,24].

To confirm our observations, ion chromatographic fingerprints representing five tastants and controls were subjected to PLS-DA classification, and class affinities for each studied sample were summarized in a confusion matrix (Figure 3B). The cellular responses towards sweet, bitter and umami stimuli must have been significant, because these classes were perfectly recognized and not misclassified with controls in all cases (100% accuracy for these three tastes). The sour tastant was correctly identified only in four out of nine cases—as many as five times it was regarded as a control. The lack of discrimination from controls was even more visible in the case of the salty taste, the chromatographic fingerprints of which were assigned incorrectly in almost all cases. The controls were also misclassified for the ionic tastants (NaCl and citric acid) in five out of 45 cases.

Taking into account STC-1 cells’ natural ability to sense only three non-ionic tastes, ion chromatographic fingerprints were classified using the PLS-DA model considering only four classes of cellular response, one for each non-ionic taste: sweet, bitter and umami, and the “no taste” samples (Figure 3C). This last class included all controls (cells not exposed to any of the stimuli) and two ionic tastants that should also have been indiscriminative for the studied chemosensory cells. The confusion matrix summarizing the recognition capability of this PLS-DA model revealed the almost perfect identification of the respective ion chromatographic fingerprints of the cellular responses. The accuracy of the classification increased from 80.0 to 98.9%, showing the almost perfect identification of all non-ionic tastes that can be sensed by STC-1 cells. This result confirms that the ability of STC-1 cells to sense three basic tastants can be coupled with ion chromatographic fingerprinting of the cellular response to provide effective taste recognition in in vitro conditions. The presented taste sensing strategy could find possible applications in all taste research, with great importance in the pharmaceutical and foodstuff industries. Moreover, chromatographic fingerprinting of the cellular response, not only for chemosensory cells, could be useful in all cell-signaling studies, as an alternative approach to traditionally applied methods.

## 4. Conclusions

We proposed a new method for in vitro taste recognition based on the ion chromatographic fingerprinting of chemosensory cells’ response to various taste stimuli. Ion chromatographic fingerprints were classified using PLS-DA, showing good discrimination of bitter, sweet and umami tastes compared to controls, and worse discrimination in the case of two other tastants, which is in good accordance with the natural ability of the applied STC-1 cells to taste sensing. The presented results are important for all studies related to the development of taste sensing systems, since natural recognition elements are involved, the chemosensory functioning of which is capable of mimicking natural taste sensation in mammals. Moreover, it was shown that ion chromatographic fingerprinting can be a good transduction method for revealing and decoding taste-specific cellular response. To the best of our knowledge, such a strategy for receiving and processing taste sensing information is presented here for the first time.

This article presents preliminary research. In the future, the correlation of ion chromatographic fingerprints with various concentration levels of taste stimulants should be studied. The more challenging task would be a comparison of the similarity of the fingerprints of the cellular response to taste stimulants with differing chemical structures, but similar taste properties. This is the subject of our ongoing research.

## Supplementary Materials

The following are available online at https://www.mdpi.com/1424-8220/19/5/1062/s1, Figure S1: Experimental workflow, Figure S2: Responses of the PLS-DA models for chromatographic fingerprints analysis in the case of (A) sour; (B) salty; (C) umami taste.
