# Supplementary material for: Ion Chromatographic Fingerprinting of STC-1 Cellular Response for Taste Sensing
Source: Sensors (Basel). 2019 Mar 2;19(5):1062. doi: 10.3390/s19051062 (PMC6427131; doi:10.3390/s19051062)
Supplement: Supplementary file 1 [file sensors-19-01062-s001.pdf]

SUPPLEMENT

Ion Chromatographic Fingerprinting of STC-1 Cellular Response for Taste Sensing

Marcin Zabadaj [1], Aleksandra Szuplewska [1], Maria Balcerzak [2], Michał Chudy [1] and Patrycja Ciosek-Skibińska [1,*]

[1] The Chair of Medical Biotechnology, Faculty of Chemistry, Warsaw University of Technology, Noakowskiego 3, 00-664 Warsaw, Poland; mzabaj@ch.pw.edu.pl (M.Z.); aszuplewska@ch.pw.edu.pl (A.S.); chudziak@ch.pw.edu.pl (M.C.)

[2] Faculty of Chemistry, Warsaw University of Technology, Noakowskiego 3, 00-664 Warsaw, Poland; mbal@ch.pw.edu.pl

* Correspondence: pciosek@ch.pw.edu.pl; Tel./fax: (+48)-22-234-7873

Received: 30 January 2019; Accepted: 26 February 2019; Published: date

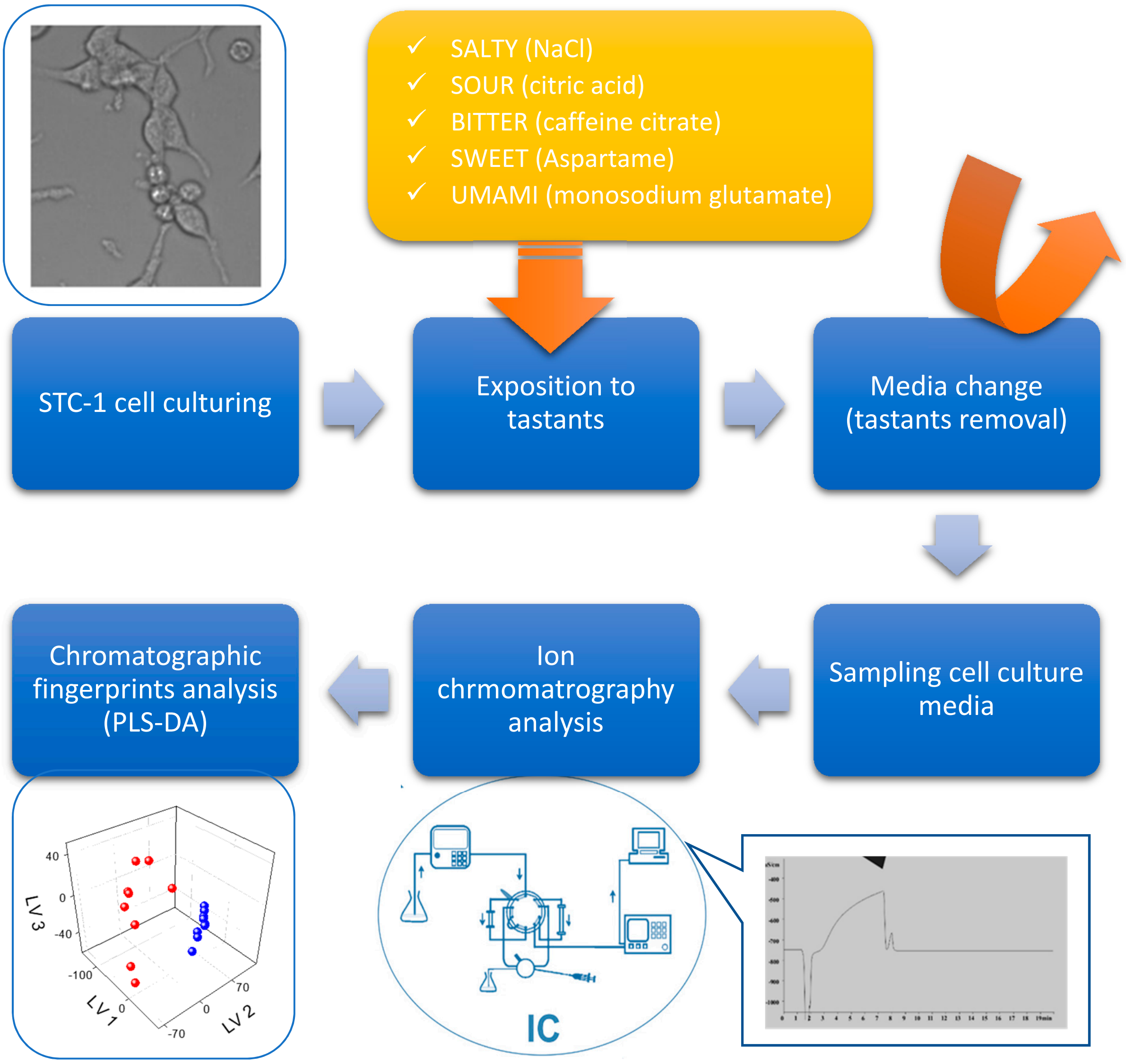

Figure S1. Experimental workflow.

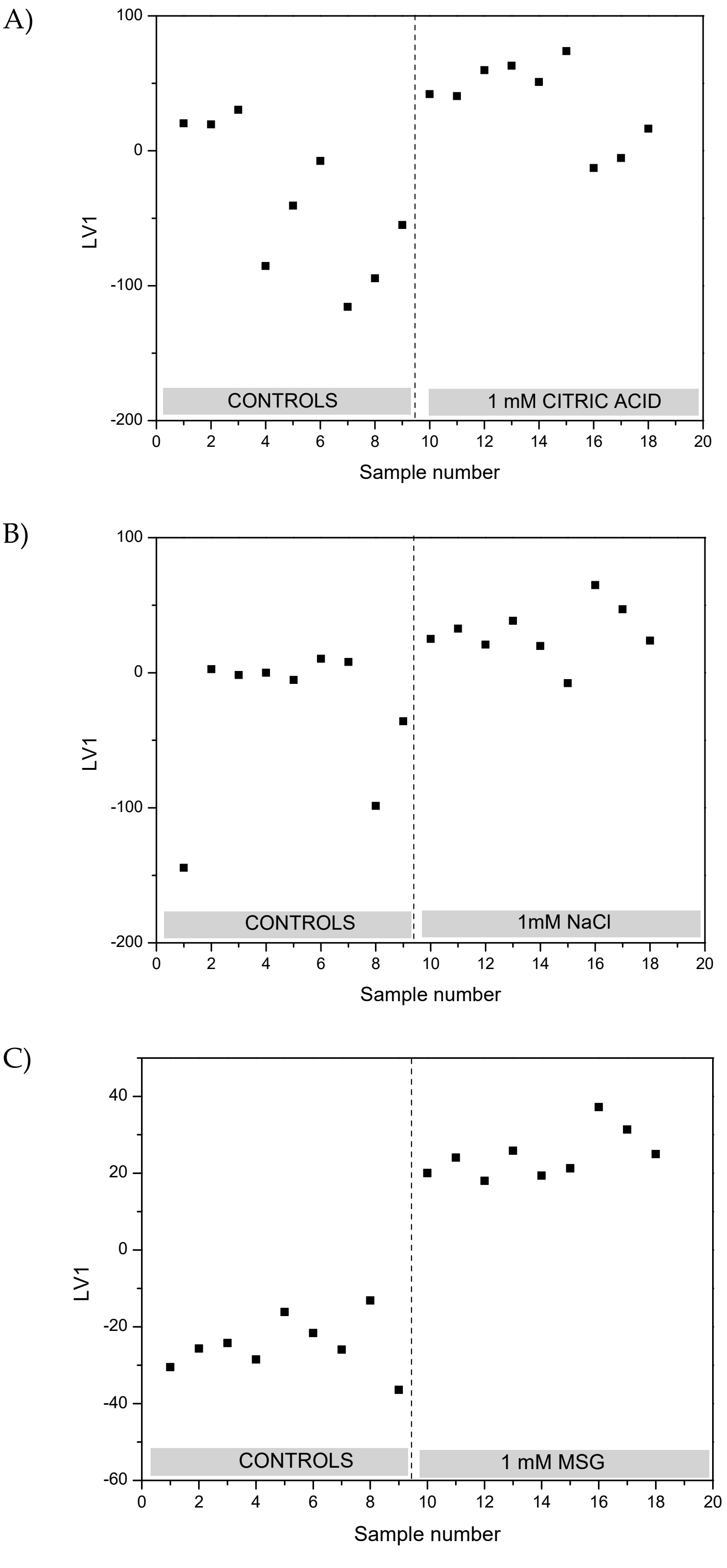

Figure S2. Responses of the PLS-DA models for chromatographic fingerprints analysis in the case of A) sour; B) salty; C) umami taste.
